# Mental Imagery in Patients with Prolonged Grief Disorder: a Comparison with Matched Bereaved Healthy Controls

**DOI:** 10.1007/s11126-021-09914-x

**Published:** 2021-03-30

**Authors:** Octavia Harrison, Claudio Wiedenmann, Rita Rosner, Regina Steil

**Affiliations:** 1 Department of Clinical Psychology and Psychotherapy, Goethe University Frankfurt, Varrentrappstr. 40-42, 60486 Frankfurt, Hesse Germany; 2 Department of Clinical and Biological Psychology, Catholic University Eichstaett-Ingolstadt, Eichstätt, Germany; 3 Center for Mind, Brain and Behavior (CMBB), University of Marburg and Justus Liebig University, Giessen, Germany

**Keywords:** Mental imagery, Visual imagery, Prolonged grief disorder, Bereavement, Phenomenology, Psychopathology

## Abstract

Mental imagery is a transdiagnostic feature that has been increasingly researched in mental disorders in the past years. This study is the first to investigate mental imagery in individuals suffering from Prolonged Grief Disorder (PGD), a new disorder which will be included into the new edition of the International Classification of Diseases and Related Health Problems (ICD-11).

Our objective was to find out to what extent patients suffering from PGD differ from healthy, but equally bereaved, controls in terms of mental imagery, and how mental imagery is related to psychopathology. Patients with PGD and matched bereaved healthy controls (*n* = 54) completed a mental imagery questionnaire specifically designed for the study, and other established measures of psychopathology. Patients suffering from PGD reported mental images more frequently, had less control over them, and described negative images as more vivid than did healthy controls. Also, in reaction to mental images, patients less frequently experienced joy, but more often grief, anger and guilt. Besides these group differences, significant correlations between mental imagery other psychopathological measures could be found. Mental imagery is clearly related to PGD. The underlying mechanisms on whether it is a developing or maintaining factor need to be addressed in future studies. Future research should also investigate in what way mental imagery might be used in therapeutic approaches.

## Introduction

The 11th edition of the International Statistical Classification of Diseases and Related Health Problems (ICD-11) introduces prolonged grief disorder (PGD) as a new diagnosis in the chapter ‘Disorders Specifically Associated with Stress’ [1, 2]. The introduction of this new diagnosis has been the subject of controversial discussions for years with the fear ofpathologizing a natural response to loss on the one hand, and the responsibility to recognize and to adequately treat a strongly dysfunctional reaction on the other [3].

During the past decades different diagnostic criteria and terminologies have been introduced, from “pathological grief” [4] to “complicated grief” (CG), “persistent complex bereavement disorder” (PCBD) [5], and eventually “prolonged grief disorder” [6]. Due to the existence of several competing diagnostic proposals, it was eagerly awaited how the diagnosis would ultimately be defined in the upcoming ICD [7]. PGD according to the ICD-11 is based on the proposal of Prigerson and colleagues [6], but differs in some points,  e.g.,  there is no specification of the number of the accessory symptoms threshold [8, 9]. In detail, according to the ICD-11, PGD can be diagnosed following the death of someone with whom an individual had a close relationship, if the individual either experiences intensive longing for the deceased or is persistently preoccupied with the deceased. These criteria must be accompanied by intense emotional pain exemplified by 10 symptoms. Furthermore, the grief response has to persist for an atypically long period of time following the loss (at least 6 months), has to clearly exceed expected social, cultural or religious norms and has to cause significant impairment in personal, family, social, educational, occupational, or other important areas of functioning [2]. Maciejewksi et al. [10] analyzed whether PGD according to the ICD-11 proposal (PGD-ICD), PGD according to Prigerson, CG and PCBD represent the same underlying construct. They employed data derived from the Yale Bereavement Study and could demonstrate that PGD-ICD, PGD and PCBD underlie the same construct, thus, only differ from another from a semantic perspective. Yet, CG appeared to be different, as it only led to moderate agreement with the other three diagnoses. According to their results the application of the PGD-ICD, PGD or the PCBD sets would each result in 10% of the individuals being diagnosed with PGD/ PCBD, whereas the application of the CG criteria would result in a prevalence rate of 30% in the same sample.

Although distressing mental images do not represent a diagnostic criterion (unlike, for example, in the case of Posttraumatic Stress Disorder (PTSD), Acute Stress Disorder, and Obsessive–Compulsive Disorder [5, 11, 12]), a majority of PGD-patients reports to have intrusive loss-related memories or images [13–16]. Typical content of these images is reenactment of death, revenge fantasies, rescue fantasies or reunion [16]. Therefore, it has been assumed that mental imagery may represent an important maintaining factor [13–16].

Mental imagery in the form of frequent, vivid and distressing images are very common in psychopathology and have already been investigated in several other mental disorders, i.a. depression, anxiety disorders, and PTSD [17–22]. In PTSD, mental images are usually related to the traumatic event or the preceding moment, signaling the onset of the event, causing feelings of guilt, anger, shame, sadness or fear [19, 23, 24]. In depression, mental imagery is often based on memories, and the content consists mainly of injury, death, and illness of a family member or close friend of the patient, but also of assault and interpersonal problems causing feelings of sadness, anger, helplessness and fear [25–27]. Very importantly, imagery appears to have a stronger impact on emotions than verbal processing, thereby acting as an emotional amplifier, which is more likely to promote behavior [20, 21, 28, 29]. This emphasizes its importance as a maintaining factor in various kinds of psychopathology, as has been demonstrated by a large body of research in the past decades [17, 21, 28, 30–35]. Another important feature of highly emotional mental imagery is that these images often appear particularly real and thus especially believable so that even if other contradictory information exists this other information is blocked and not integrated [20]. For most kinds of psychopathology mental imagery is associated with distress or negative emotions (e.g., in PTSD intrusion about a bike accident) and often results in avoidance behavior (not going by bike anymore) [20]. However, as research on addictive behavior has demonstrated [36], positive imagery can also be maladaptive (e.g. deliberately imagining about the substance), as it can result in negative emotions and increase craving, eventually causing a relapse.

Based on these findings, mental imagery has been identified as a transdiagnostic feature and targeted in therapeutic interventions. Hackmann and Holmes [19], for example, refer to techniques such as imaginal exposure and systematic desensitization, imagery reduction via competition (interference by performing another task), imagery rescripting and positive imagery retraining, as in cognitive bias modification training. The effectiveness of such methods has been documented in many studies [30, 37–42]. Another idea was put forward by Holmes and Mathews [20] and Blackwell [28]. They support the idea that individuals affected by mental imagery need to be informed about the mechanisms of imagery and realize that *images are just images and not reality* and apply meta-cognitive or mindfulness-based approaches [20, 28, 43, 44]*.*

Given the fact that PGD represents a relatively new diagnostic category, and the agreement upon diagnostic criteria has been lacking in the past, there are almost no studies investigating mental imagery in PGD systematically. The only studies having investigated mental imagery in grief, have assessed treatment seeking mourners, who, however, did not necessarily had to fulfill the diagnostic criteria for PGD [13, 16]. Also, neither study used a control group. In their study, Boelen and Huntjens [13] could show that intrusive images are common among mourners. Positive images of the deceased were reported most frequent by more than 60%. Also, it was shown that these kinds of images were related with CG, but not with depression and anxiety. Furthermore, high levels of re-enactment fantasies, negative images of the future, and intrusive images of moments surrounding the death were correlated with higher levels of CG, depression, and anxiety. In a later study by Baddeley et al. [16], more than 90% of the treatment-seeking sample reported to have some kind of grief-related imagery. Most frequently, the images contained reenactment, reunion, or remorse. The frequency of the examined images was positively associated with CG symptoms, depressive symptoms and PTSD symptoms. Especially imagery about reunion can be assumed to foster symptoms of yearning and longing [16]. The authors have even postulated that some images might lead to a fixation. The reported findings are in line with earlier publications, which studied intrusive imagery in survivors of violent death [45, 46]. In these older studies participants exhibited mainly a PTSD symptomatology and therefore these results cannot easily be generalized for PGD. Consequently, knowledge about different kinds of mental imagery in individuals diagnosed with PGD is scarce. However, knowing about the general occurrence of imagery in PGD and its peculiarities might promote a better understanding of the disorder and underlying psychological mechanisms and eventually could bring in new ideas when it comes to treatment [13].

The aim of the subsequently presented study is to specifically investigate mental imagery in PGD in a sample of patients suffering from PGD (experimental group, EG) as compared to an equally bereaved matched healthy control group (CG). For that purpose, we have specifically designed a mental imagery questionnaire, which will be described in detail in the methods section.

The first aim of our study is to investigate the frequency of different kinds of imagery and their associations with different measures of psychopathology. Based on earlier findings [13, 16, 34, 47, 48], we hypothesize that patients suffering from PGD report mental imagery more frequently than healthy bereaved control participants. Further, we assume that the frequency of mental images is positively associated with symptoms of grief, depression and general mental distress.

The second aim of our study is to investigate the impact vividness and controllability of imagery have on symptom severity. Based on previous research [21, 49–51], we expect patients diagnosed with PGD to have less control and experience images as more vivid in comparison to healthy bereaved control participants.

Lastly, one aim is to explore different aspects of imagery that had not been researched before. This aim includes what emotions are elicited by the images, whether they are based on memories or are fictious, and, eventually, sensory qualities of the images.

## Materials and Methods

### Participants and Procedure

Participants belonging to the experimental group (EG) were bereaved individuals who sought and received therapeutic treatment as a part of a German-wide multicenter randomized controlled trial at the Center for Psychotherapy in Frankfurt [52]. The aim of the main study was to investigate the effectiveness of integrative cognitive behavior therapy for PGD (PG-CBT) [15, 53] in comparison to an active control condition, i.e., Present-Centered Therapy (PCT) [54].

The EG included individuals between 18 and 75 years of age, who meet the diagnostic criteria for PGD according to the *Interview for Prolonged Grief-13* (PG-13) [55]. Also, PGD had to be the primary diagnosis. Further inclusion criteria were sufficient knowledge of German, adequate cognitive skills and signed consent. Acute psychotic disorder, major substance-related disorder, acute suicidal tendencies, other psychotherapeutic treatment, irregular antidepressant medication, regular use of benzodiazepines, antipsychotics or opiates, or participation in a further intervention study led to exclusion.

For the control group (CG), subjects were recruited, who could be compared to the EG with regard to age, gender and time elapsed since the loss. The main inclusion criterion was the death of a significant other at least 6 months ago. The presence of PGD according to PG-13 or any other mental disorder according to the German version of the *Structured Clinical Interview for DSM-IV* (SCID-I) [56] past or present, led to exclusion. Further inclusion criteria were again sufficient knowledge of German, adequate cognitive skills and signed consent. Both groups were recruited through professional/ self-referral, information events at the clinic, email distribution lists, notices in public areas, and social media. The data were collected between June 2018 and February 2020.

### Measures

*Sociodemographic data* and *loss-related variables* were obtained in a semi-structured manner by a trained interviewer, as were the PG-13 and the SCID-I. All other measures presented are self-report measures completed by the participants.

An extended German Version of the *Interview for Prolonged Grief-13* (PG-13) [55, 57] was employed to assess PGD. The interview consists of 13 items and allow a PGD diagnosis according to the consensus criteria proposed by Prigerson et al. [6]. When the study was conceptualized, the prospective criteria of PGD according to the ICD-11 had not been officially introduced yet, we therefore decided to use the stricter consensus-criteria by Prigerson et al. [6]. To diagnose PGD, five criteria must be met: Loss of a significant other (criterion A), at least daily experienced separation distress (criterion B), cognitive, emotional, and behavioral symptoms (criterion C), a duration of the separation distress symptoms for at least 6 months (criterion D), and a significant functional impairment in different domains (criterion E) [6]. A value for PGD symptomatology can be calculated by summing up eleven symptom-related items which are rated on a 5-point Likert scale. Studies using the original PG-13 indicate good psychometric properties with an internal reliability of α = 0.82 [6]. A high Cronbach’s α of .96 was found in our sample.

In addition to the PG-13, the German version of the *Inventory of Complicated Grief* (ICG-D) [58, 59] was used to assess PGD. The questionnaire consists of 19 items and has to be answered on a 5-point Likert-scale. A sum score of >25 is considered to indicate clinical levels of complicated grief [59]. The internal consistency is comparably high in both the English (Cronbach’s α = .94) and the German version (Cronbach’s α = .87) [58, 59]. The internal consistency for our sample was also high with Cronbach’s α = .98.

To assess the severity of depression the German translation [60, 61] of the *Beck Depression Inventory-II* (BDI-II) was used. This questionnaire consists of 21 self-report items rated on 4-point Likert scale. The internal reliability was found to be good for an American sample (.86 < α < .92) [62], as well as for German samples (.89 < α < .93) [63]. For our sample we obtained a high internal consistency of α = .97.

The German version of *Brief Symptom Inventory* (BSI) [64, 65] was used to measure general mental distress. The BSI consists of 53 items caused by psychological or somatic symptoms over the past seven days using a 5-point Likert scale. For our analyzes we used the Global Severity Index (BSI-GSI) which measures the perceived level of distress. In the norm sample very high internal consistencies of α = .92 to α = .95 were determined [64]. Comparable results were obtained in our sample, where α = .98.

To assess mental imagery, a questionnaire (*mental imagery questionnaire*) [66] was constructed. Already in 2015, one of the authors (RS) had conducted a study on mental imagery [67]. Again, the structure of the questionnaire was based on items in the Intrusion Interview and Intrusion Questionnaire by Hackmann et al. [47]. First, a description of mental imagery in general was provided. Then, participants were asked about eight types of images in detail. These images were positive and negative image about the deceased, positive and negative loss-related images of the bereaved him−/herself, positive and negative image of the own future and finally positive and negative image of the own future including the deceased. Participants should indicate how often they experienced each specific type of imagery on a 5-point Likert scale ranging from 1 (never) to 5 (several times a day). Subsequently, we asked the participants to describe one picture in detail in an open format (e.g., instruction: “What is the most positive or most often experienced positive image about?”). Participants had to answer a specific set of questions with respect to that image. The first question referred to whether the image was based on a real memory or whether it was fictitious. As in the earlier study [67], we included imagery characteristics as vividness and controllability. Both were rated on a 11-point Likert scale ranging from 0 (not at all) to 10 (extremely). In addition to that, we assessedemotions elicited by the image (either joy, relief, anger, fear, disgust, sadness, and/ or guilt), as well as sensory qualities (i.e. seeing, hearing, smell, taste and touch) involved. Multiple nominations were possible.

### Statistical Analyses

The SPSS Software package (Version 27, IBM Corp., Armonk, NY, USA) was used to document and analyze the data collected. In order to ensure comparability of the data collected, it was first checked whether both groups differed significantly with regard to socio-demographic variables as well as loss-related variables. For variables that are measured on a nominal or ordinal scale level, χ2- tests were conducted. When at least five response grades were present, t-tests for independent samples were calculated [68]. As a correlative measure the product-moment correlation coefficient according to Pearson was calculated. Both, t-test and the correlation coefficient of the product-moment correlation according to Pearson, have proven to be robust against violation of the normal distribution assumption [69, 70]. Therefore, in the few cases where normal distribution was violated (time since loss, fictions score), we nevertheless used parametric statistics. Unless otherwise stated, the results are presented at the significance level of α = .05. The method of mean imputation was used for the BDI, BSI-GSI, PG-13, and ICG-D when data were missing, and an item was left out.

## Results

### Sample Characteristics

Both groups consisted of 27 subjects each and together had an average age of *M* = 55.80 (*SD* = 13.08) years. The majority of the participants (92.59%) was born in Germany and had a university-entrance qualification (72.22%). In none of the demographic variables a significant group difference could be found. On average, 42.91 months had passed since the loss. In the EG the loss of a partner was reported most frequently (*n* = 10), whereas in the CG the death of a parent was reported most often (*n* = 11). Nevertheless, there was no significant difference between the two groups in this and most other variables surveyed. The groups differ only in terms of age of the deceased (*t* = 2.58, *p* = .013), whereby the deceased in the CG were on average about 16 years older. All of the sociodemographic and loss-related variables are reported in Table 1.

**Table 1:** Sociodemographic and loss-related variables of both groups

	EG	CG	Test Statistics
Demographics			
Age: *M (SD)*	55.59 (12.25)	56.00 (13.90)	*t = 0.11; p = .909*
Gender			
Female	24	22	*χ2 = 0.59; p = .444*
Male	3	5	
Loss-related variables			
Age of the deceased (*M; SD*)	54.26 (21.58)	70.11 (24.23)	*t = 2.58; p = .013*
Gender of the deceased			
Female	8	13	*χ2 = 1.95; p = .163*
Male	19	14	
Kinship			*χ2 = 7.95; p = .159*
Own child	5	2	
Partner	10	6	
Parent	7	11	
Siblings	2	0	
Another family member	2	7	
Friend	1	1	
Time since loss in months (*M; SD*)	35.52 (59.48)	50.30 (56.87)	*t = 0.933; p = .355*
Cause of death			*χ2 = 5.69; p = .128*
Disease	23	22	
Violent crime	1	0	
Traffic accident	1	5	
Suicide	2	0	
Expectability of death			*χ2 = 2.70; p = .100*
Expectable	9	15	
Surprisingly	18	12	

*EG* experimental group (*n* = 27), *CG* control group (*n* = 27)

Finally, the groups were compared with regard to psychopathology. The results, presented in Table 2, revealed significantly higher values for the EG in all areas. Thus, the results confirm that the EG was indeed seriously affected in terms of mental health, both in absolute terms and in relative terms compared to the CG.

**Table 2:** Means and group differences for psychopathology measures

	EG	CG	Test Statistics
ICG-D *(M; SD)*	45.88 (8.05)	8.23 (6.92)	*t* = −18.44; *p* = <0.001
PG-13 *(M; SD)*	42.30 (4.72)	16.04 (5.18)	*t* = −19.47; *p* = <0.001
BDI-II *(M; SD)*	30.97 (8.30)	3.19 (3.06)	*t* = −16.33; *p* = <0.001
BSI-GSI *(M; SD)*	1.50 (.63)	.20 (.14)	*t* = −10.44; *p* = <0.001

*EG* experimental group (*n* = 27), *CG* control group (*n* = 27), *BDI-II* Beck Depression Inventory-II, *BSI-GSI* Brief Symptom Inventory, *PG-13* Interview for Prolonged Grief-13, *ICG-D* Inventory of Complicated Grief

As outlined in the materials and method section, PGD had to be the primary diagnosis. However, secondary diagnoses were allowed and 19 of our 27 (70.3%) participants fulfilled the criteria for at least one other diagnosis. All of them either fulfilled the criteria for depression (*n* = 18) or dysthymia (*n* = 2), or both. Five cases fulfilled the criteria for two additional diagnoses, one for three and one for four. The diagnoses fulfilled were as follows: agoraphobia and panic attacks (*n* = 3), somatization (*n* = 2), specific phobia (*n* = 2), social anxiety disorder (*n* = 1) and eating disorder (*n* = 1).

### Mental Images

Average values for frequency, vividness and control over mental images were determined separately for all positive and all negative images. Since multiple testing in the same sample would lead to an alpha error accumulation and due to the fact that it can be assumed that time since loss is a source of variation that affects the dependent variables (e.g. the longer the time since death, the less often mental images of the deceased are experienced), a Multivariate Analysis of Covariance (MANCOVA) was performed. Since some individuals mentioned that they never had a mental image, we calculated a separate MANCOVA for frequency considering all 54 subjects, and another MANCOVA only considering those persons who had indicated that they had experienced mental images for the variables vividness and control. The first MANCOVA on frequency revealed significant differences between both groups after the effect of the time-since-loss was controlled for (Pillai’s trace value = 0.679, *p* = <0.001), but no effect of the time since loss (Pillai’s trace value = 0.011, *p* = .762). Since Pillai’s trace value was significant, each dependent variable was tested with the univariate F tests. Post-hoc comparisons revealed the EG reported to experience positive and negative mental images with a significantly higher frequency. However, when looking at the images separately, results are still all significant, but when it comes to positive images about the own future the CG reported this kind of image significantly more often than the EG. Frequencies for every separate image are shown in Table 3.

**Table 3:** Endorsement and mean scores of intrusion items

Type of image		Endorsement *n* (%)					M (SD)	Test Statistics
	Group (*n*)	Never (value 1)	once a month (value 2)	once a week (value 3)	once a day (value 4)	several times a day (value 5)		
Positive image of the deceased	EG (27)	–	4 (14.8)	4 (14.8)	8 (29.6)	11 (40.7)	3.96 (1.09)	*t* = −5.21; *p* < .001
	CG (27)	6 (22.2)	8 (29.6)	8 (29.6)	5 (18.5)	–	2.44 (1.05)	
Negative image of the deceased	EG (26)	2 (7.4)	4 (14.8)	6 (22.2)	5 (18.5)	9 (33.3)	3.58 (1.33)	*t* = −7.61; *p* < .001
	CG (27)	18 (66.7)	7 (25.9)	2 (7.4)	–	–	1.41 (.64)	
Positive loss-related image of oneself	EG (26)	7 (25.9)	6 (22.2)	5 (18.5)	5 (18.5)	3 (11.1)	2.65 (1.38)	*t* = −2.33; *p* < .05
	CG (27)	10 (37.0)	9 (33.3)	8 (29.6)	–	–	1.93 (.93)	
Negative loss-related image of oneself	EG (26)	1 (3.7)	3 (11.1)	5 (18.5)	11 (40.7)	6 (22.2)	3.69 (1.09)	*t* = −9.75; *p* < .001
	CG (27)	20 (74.1)	5 (18.5)	2 (7.4)	–	–	1.33 (.62)	
Positive image of the own future	EG (27)	11 (40.7)	11 (40.7)	2 (7.4)	3 (11.1)	–	1.89 (.97)	*t* = 2.45; *p* < .05
	CG (27)	6 (22.2)	7 (25.9)	8 (29.6)	2 (7.4)	4 (14.8)	2.67 (1.33)	
Negative image of the own future	EG (27)	2 (7.4)	2 (7.4)	4 (14.8)	10 (37.0)	9 (33.3)	3.81 (1.21)	*t* = −8.33; *p* < .001
	CG (27)	12 (44.4)	13 (48.1)	2 (7.4)	–	–	1.63 (.63)	
Positive image of the own future, which includes the deceased	EG (21)	9 (33.3)	6 (22.2)	2 (7.4)	3 (11.1)	1 (3.7)	2.10 (1.26)	*t* = −2.14; *p* < .05
	CG (27)	17 (63.0)	7 (25.9)	3 (11.1)	–	–	1.48 (.7)	
Negative image of the own future, which includes the deceased	EG (21)	6 (22.2)	3 (11.1)	2 (7.4)	5 (18.5)	5 (18.5)	3.00 (1.61)	*t* = 5.03; *p* < .001
	CG (27)	23 (85.2)	2 (7.4)	1 (3.7)	1 (3.7)	–	1.26 (.71)	

In the second MANCOVA, aiming at investigating vividness and control revealed significant differences between both groups after the effect oftime-since-loss was controlled for (Pillai’s trace value = 0.700, *p* = <0.001) but no effect of time-since-loss (Pillai’s trace value = 0.145, *p* = .380). Again, each dependent variable was tested with univariate F tests. The EG reported significantly more often to have less control over mental images, and they described negative mental images as more vivid, but this was not the case for positive images. In no case, a relation with time since loss was detected. The results for both MANCOVAs are shown in Table 4.

**Table 4:** Mean values for frequency, control and vividness for positive and negative mental images separately reported with time since loss as covariates

	EG	CG	Group			Time Since Loss		
	*M (SD)*	*M (SD)*	*F*	*p*	𝜂^2^	*F*	*p*	𝜂^2^
Frequency pos.	2.67 (0.82)	2.13 (0.74)	6.24	.016	.109	0.01	.978	.000
Frequency neg.	3.54 (0.95)	1.41 (0.48)	104.53	<0.001	.672	0.45	.506	.009
Control pos.	5.17 (2.81)	8.17 (2.34)	14.65	<0.001	.250	2.38	.130	.051
Control neg.	3.20 (2,07)	6.91 (2.67)	27.30	<0.001	.383	0.93	.341	.021
Vividness pos.	7.45 (1.89)	6.47 (1.94)	3.53	0.067	.074	1.63	.208	.036
Vividness neg.	7.99 (1.98)	5.13 (2.79)	17.83	<0.001	.288	1.70	.199	.037

*Pos*. positive imagery, *Neg*. negative imagery. *N* for frequency pos. and frequency neg. = Experimental group: *n* = 27, control group: *n* = 27. For the remaining variables sample size is Experimental group: *n* = 25, control group: *n* = 22

Another MANCOVA was conducted to check whether the groups differed in the frequency with which certain emotions were elicited by the mental images. When screening the data, we noticed that disgust was only indicated by one person. Thus, this variable shows no (co)variance and was therefore excluded from the analysis. Again, time since loss was controlled for. The MANCOVA revealed significant differences between both groups after the effect of time-since-loss was removed (Pillai’s trace value = 0.50, *p* = <0.001). Again, no connection with time since loss could be established (Pillai’s trace value = 0.09, *p* = .593). No difference was found in post-hoc comparisons between the groups regarding the emotions relief and fear, whereas in contrast grief, guilt and anger were triggered significantly more often and joy less often by mental imagery in the EG. Results are shown in Table 5.

**Table 5:** Frequency of certain emotions triggered by mental images (without disgust) with time since loss as covariate

	EG	CG	Group			Time Since Loss		
	*M (SD)*	*M (SD)*	*F*	*p*	𝜂^2^	*F*	*p*	𝜂^2^
Joy	.25 (.23)	.49 (.24)	14.35	<0.001	.223	0.12	.730	.002
Relief	.15(.14)	.16 (.84)	0.01	.907	.000	1.70	.198	.033
Anger	.25 (.24)	.04 (.09)	15.58	<0.001	.238	0.07	.794	.001
Fear	.23 (.21)	.15 (.14)	2.29	.137	.044	0.01	.943	.000
Grief	.64 (.29)	.28 (.25)	23.52	<0.001	.320	2.40	.128	.046
Guilt	.36 (.31)	.11 (.13)	13.88	<0.001	.217	0.51	.480	.010

*EG* experimental group (*n* = 27), *CG* control group (*n* = 26)

A chi-square-test was used to compare, if fictitious or real-memory images occurred to a different extent in the two groups. This comparison was made for each type of mental image, resulting in 8 comparisons. In order to avoid alpha cumulation, the alpha-error level was adjusted to 0.05/8 = 0.00625 with the Bonferroni correction [70]. No difference between the groups was found. Moreover, a mean score over all types of mental images was calculated. If an image was based on a real memory it was encoded with 0. If it was fictious it was encoded with 1. The resulting values were *M* = 0.27 (*SD* = 0.17) for the CG and *M* = 0.30 (*SD* = .22) for the EG. Thus, 30% of the mental images reported by the EG are fictitious and 70% are based on real memories. A group comparison showed no significant difference (*t* = −0.50, *p* = .623). Therefore, the groups do not differ in whether mental images are based on real memories or fiction.

Another aim was the exploration of sensory qualities being involved in mental imagery. A MANCOVA could not be calculated in this case, because this approach only considers complete observations (information on the number of sensory qualities present for all images). This was only the case for 15 participants. This sample size does not allow for meaningful interpretation. In order to use all information available, eight individual group comparisons were calculated for each type of mental image. Again, a Bonferroni correction of the significance level was performed, but no group difference was detected.

The relationships between characteristics of mental imagery and psychopathology values were calculated with the Pearson’s product-moment correlation. In addition to these variables of psychopathology, the connection between time since los*s* and mental imagery characteristics was assessed. Results are presented in Table 6.

**Table 6:** Pearson’s product-moment correlation for psychopathology values and time since loss with characteristics of and emotions triggered by mental imagery

	**frequency**	**vividness**	**control**	**joy**	**relief**	**anger**	**grief**	**fear**	**guilt**	**disgust**
**PG-13**	.736**	.509**	−.625**	−.452**	−.059	.548**	.599**	.162	.430**	.135
**ICG-D**	.715**	.566**	−.631**	−.454**	−.034	.456**	.605**	.208	.465**	.134
**BDI**	.682**	.479**	−.580**	−.441**	.040	.506**	.522**	.190	.375**	.225
**BSI-GSI**	.651**	.449**	−.551**	−.367**	.033	.497**	.494**	.195	.280*	.238
**Time since loss**	−.127	.135	.214	−.001	.184	−.011	−.226	−.009	.048	−.013

*p < .05 (2-sided). ** *p* < .01 (2-sided); *n* ranging from 52 to 54. *BDI-II* Beck Depression Inventory-II, *BSI-GSI* Brief Symptom Inventory, *PG-13* Interview for Prolonged Grief-13, *ICG-D* Inventory of Complicated Grief

As a result, values for psychopathology were found to be all highly positively correlated with the frequency of mental images (.651 < *r* < .736), medium to highly correlated with their vividness (.479 < *r* < .566), and medium to highly negatively correlated with control over them (−.551 < *r* < −.631). In addition, psychopathology values correlated medium with joy (−.441 < *r* < −.454), medium to highly with anger (.456 < *r <* .548), highly with grief (.522 < *r* < .605) and medium with guilt (.280 < *r* < .465). At the same time, no connections with the other, non-grief specific emotions (relief, fear and disgust) could be identified. In no case a connection between time since loss and mental imagery could be observed.

## Discussion

This study is the first to examine mental imagery in a sample of patients suffering from PGD in contrast to equally bereaved healthy controls.

The first aim of our study was to investigate the frequency of different mental images and their associations with different measures of psychopathology. As expected, the results confirmed that the groups differ significantly in terms of frequency of mental images, with the EG more frequently reporting positive and negative ones. However, there is one exception, positive images about the own future are reported significantly more often by the CG than the EG. We also found that different kinds of psychopathology (PGD measured by the ICG-D and the PG-13, depression measured by the BDI-II, and general mental distress measured by the BSI) were significantly associated with the frequency of mental images.

The second aim of our study was to investigate vividness and controllability of imagery. In line with our hypotheses, the CG had significantly more control over the images than the EG, and the EG perceived negative mental images significantly more vivid that the CG. As for frequency, significant associations of controllability and vividness with psychopathology were found.

In our exploratory analysis, we revealed that the EG experienced significantly more often grief, anger, and guilt, but less often joy in reaction to mental imagery. However, no significant differences could be obtained for fear and relief. Finally, psychopathology was significantly associated with the emotions joy, anger, grief, and guilt following mental images. Neither in the group comparisons nor in the correlations could we detect an effect due to time since loss, indicating that mental images are not a transient phenomenon directly following a death, but persist over time. Also, no difference could be found neither concerning the ratio of how often mental images were based on real memory or on fiction, nor for the number of sensory qualities being involved in the images. In addition, like in depression, PTSD and many anxiety disorders [19, 26, 31, 71], we noted that the majority (in our study 70%) of mental images in PGD are based on real memories.

When considering imagery as *emotional amplifier* it makes sense that mental imagery in general, independent from its valence, can be observed more frequently and in some cases more vividly in individuals suffering from PGD, since PGD is also characterized by intense emotional pain. We argue that imagery might represent one important source for this symptom, making it even more important to address mental imagery in therapy. Given the results of research on PTSD and depression [19, 23–27] the higher frequency of negative images, especially with respect to images of the deceased and images of the own future appears reasonable. In line with the cognitive-behavioral model of CG [72] especially negative appraisals about the future are a crucial maintaining factor. However, the fact that positive images about the deceased are reported at least on a daily basis by more than 70% is interesting. Previous research has indicated that some intrusions might actually represent “comforting portrayals of the deceased” [4] (p.270). Sometimes images might even be retrieved deliberately by the bereaved for comfort and feeling close [73]. However, as has been demonstrated for addiction [36], these primarily positive images tend to be maladaptive, because the individual then must realize that the desired object (in PGD the beloved person and in addiction the substance) is not available and actually lost for good. Thus, the positive image eventually becomes a painful reminder of what is missing, resulting in intense yearning and longing for the deceased [16].

### Limitations and Future Research

Even though our study can make a substantial contribution by shedding some light on mental imagery in PGD, it suffers from some limitations and for that purpose our results need to be interpreted with caution.

A very first limitation is our sample. First, due to our very strict criteria for inclusion, the sample size is relatively small. Especially the fact that individuals had to be diagnosed according to the PGD consensus criteria by Prigerson et al., has been a major limitation when it came to the recruitment of participants for the EG. This circumstance entailed further limitations. More than 85% of the subjects were women, a disproportionately large share had an university entrance qualification (*n* = 39, i.e., 72.2%), and all but 4 participants were born in Germany. Drawing conclusions from the data to male patients or members of other cultures is therefore not possible. Consequently, a replication of this study with more patients from the male gender, other parts of society, or other cultures would be promising. Also, we intended to match equally bereaved healthy controls, but due to time constraints and other limitations just mentioned, it was not possible to match the groups to a 100%.

A second limitation exists in our self-designed mental imagery questionnaire. Since this questionnaire is self-designed, there is no validation, and no statements can be made about psychometric properties. Also, the questionnaire is a self-report measure. Therefore, we cannot guarantee that mental imagery has been reliably and validly assessed. Especially the external validity is subject to uncertainty. Although all subjects were given a written explanation before filling out the questionnaire as to what mental images are, it is possible that some participants report rather verbal thoughts and transform them into image-like cognitions for compliance reasons.

Besides some limitations, the study nevertheless has some strengths. First, all participants have gone through a detailed diagnostic process. This process ensured that all individuals in the EG meet the diagnostic criteria for PGD, whereas for the CG it was ensured that there was no previous mental illness. Thanks to strict inclusion and exclusion criteria, a high internal validity can be assumed.

Second, literature has shown that time since loss is related to symptom severity and mental imagery. We have addressed this fact and have included this factor in our analyses. As the effect of time since loss has been adjusted, the differences found can be attributed to the groups themselves.

Third, although the lack of validation is a limitation of our self-constructed mental imagery questionnaire its comprehensiveness represents a major strength. Earlier studies employed questionnaires with only 4 or 5 items on mental imagery [13, 16]. Apart from frequency and content, we also assessed controllability, vividness, whether an image was based on real memory or on fiction, which sensory modalities were involved, and which emotions were elicited by an image. In this way our questionnaire does justice to the multifaceted nature of mental imagery and allows a deeper understanding of this phenomenon.

Altogether, our study results have important implications. We provide first empirical evidence that PGD-patients differ from healthy bereaved individuals in terms of mental imagery. As for other mental disorders, where symptom severity was shown to be positively correlated with frequency and vividness of mental images and negatively with the perceived controllability [34, 47–51], we were able to replicate these findings for PGD. The concept of imagery acting as an *emotional amplifier*, is supported by the fact that images in PGD are experienced more frequently, negative images more vividly and that images are significantly more often associated with grief, anger, and guilt. In fact, imagery could work as maintaining factor in PGD resulting in intense emotional pain, which in turn could again lead to loss-related images more often, resulting in a self-reinforcing process and eventually to *prolonged* grief. Thus, mental images can also contribute to the development of PGD. Therefore, our findings emphasize the need to address mental imagery more appropriately in treatment. So far some already known approaches have been adapted to PGD, as for example imaginal exposure or other imaginative exercises, e.g., imagined conversation with the deceased [14, 15, 74–76]. However, a transfer of other, already existing, treatment options, also considering mental imagery, e.g. modification of negative images by questioning or challenging them, the use of meta-cognitive or mindfulness-based approaches, or the promotion of positive imagery [20, 35, 41, 43, 44, 77], to PGD could further stimulate research.

Nevertheless, before research should focus on the adaption of already known approaches, or the development of new ones, it is important to explore and evaluate mental imagery in PGD further. Studies with larger and more diverse samples, i.e., including male participants and members from other cultures, should be conducted to replicate our findings. Also, research should be expanded to other pending aspects, e.g., investigating further subgroups (kinship, type and cause of loss).  In addition to that, qualitative analyses of the exact content of mental images appears interesting. It would also be desirable to develop and validate a questionnaire for mental imagery. At the same time, similar to the work of Bonanno et al. [78], future studies analyzing the trajectories of grieving, should include the assessment of mental imagery. It would be conceivable to capture mental images bereaved individuals experience immediately after the death of a loved one, and then at later points in time during the mourning process. This design may help to further find out whether mental imagery contributes not only to the maintenance but also to the development of PGD.

## Conclusion

So far, only very few studies have investigated the impact of mental imagery in PGD. This is the first study to do so in a sample of patients suffering from PGD in comparison to equally bereaved healthy controls. The reported results emphasize the importance of mental imagery in PGD, which has been neglected for a long time. Positive as well as negative mental imagery is significantly more frequent in PGD. Positive imagery can be maladaptive and turn into the intense yearning and longing for the deceased, thereby acting as maintaining factor. Future studies targeting the treatment of PGD must integrate interventions reliably decreasing the frequency, vividness and distress caused by mental imagery.

## Data Availability

The datasets used and analyzed during the current study are available from the corresponding author on reasonable request.
